# Efficacy and Safety of Histamine H3 Receptor Antagonist/Inverse Agonist Including Betahistine for Schizophrenia: A Systematic Review and Meta‐Analysis

**DOI:** 10.1002/npr2.70034

**Published:** 2025-06-26

**Authors:** Yasufumi Nishii, Kenji Sakuma, Shun Hamanaka, Nakao Iwata, Taro Kishi

**Affiliations:** ^1^ Department of Psychiatry Fujita Health University School of Medicine Toyoake Aichi Japan

**Keywords:** betahistine, histamine H3 receptor antagonist or inverse agonist, pitolisant, schizophrenia, systematic review and meta‐analysis

## Abstract

**Aim:**

Whether histamine H3 receptor antagonists (H3R‐ANTs)/inverse agonists (H3R‐IAs) provides benefit for the treatment of schizophrenia remains unclear. This meta‐analysis was conducted to address the above clinical question.

**Methods:**

Cognitive Function Scale's composite score (primary), seven domains of cognitive function (speed of processing, attention/vigilance, working memory, verbal learning, visual learning, reasoning/problem solving, and social cognition) score, University of California San Diego Performance‐Based Skills Assessment score, psychopathology scales score, discontinuation rate, and incidence of individual adverse events were among the study outcomes. The standardized mean differences (SMD) or risk ratios (RR) with 95% confidence intervals (CIs) were calculated.

**Results:**

Our meta‐analysis included 11 double‐blind, randomized, placebo‐controlled trials (*n* = 754). Our study evaluated ABT‐288, betahistine, betahistine+reboxetine, GSK239512, MK‐0249, and pitolisant. Betahistine has an H1‐receptor agonistic reaction and H3‐receptor antagonistic reaction, while other drugs only have an H3‐receptor antagonistic/inverse agonistic reaction. Hence, we conducted a meta‐analysis for all outcomes divided by betahistine or other pooled H3R‐ANTs/H3R‐IAs. The study results show that betahistine outperformed placebo in the improvement of overall cognitive symptoms (SMD [95% CI] = −0.61 [−1.03, −0.18]), speed of processing (−0.44 [−0.87, −0.02]), attention/vigilance (−0.43 [−0.85, −0.01]), working memory (−0.48 [−0.90, −0.06]), verbal learning (−0.62 [−1.04, −0.19]), visual learning (−0.57 [−1.00, −0.15]), and betahistine+reboxetine was superior in the improvement of depressive symptoms (−4.04 [−5.10, −2.97]). Pitolisant outperformed placebo in depressive symptom improvement (−3.24 [−4.22, −2.26]). However, the results were derived from one betahistine, betahistine+reboxetine, or pitolisant study. Other pooled H3R‐ANTs/H3R‐IAs revealed risk of insomnia (RR [95% CI] = 2.18 [1.05, 4.55]). However, no differences were observed in other any outcomes between betahistine or other pooled H3R‐ANTs/H3R‐IAs and placebo.

**Conclusions:**

Some H3R‐ANTs/H3R‐IAs might provide benefit for the treatment of cognitive symptoms and depressive symptoms in individuals afflicted with schizophrenia.

## Graphical abstract

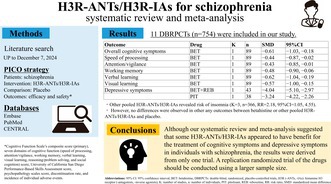

## 1 Introduction

One of the common mental illnesses with a lifetime prevalence of approximately 1% is schizophrenia [1]. Symptoms of schizophrenia are classified as positive, negative, and cognitive symptoms [2]. Antipsychotic pharmacotherapy is the primary treatment for schizophrenia, and its efficacy in treating psychopathologies is relatively well established, particularly regarding positive and negative symptoms [3]. The study analyzed recent data; the US economic burden estimate for schizophrenia reported that excess indirect costs (unemployment, productivity loss, premature mortality, and caregiving) accounted for 73.4% ($251.9 billion) of the total economic burden of schizophrenia [4]. The indirect costs might be associated with cognitive impairments [4]. However, antipsychotics fail to effectively address cognitive impairments [5]. Although our meta‐analysis showed that memantine might have benefits for the improvement of cognitive symptoms in schizophrenia, the evidence level was low [6]. Thus, only antipsychotic treatments are often considered insufficient to enhance the patients' quality of life, as most individuals tend to have a chronic course owing to various residual schizophrenia symptoms, particularly cognitive symptoms [7]. Hence, the development of novel treatments underscoring improving cognitive symptoms is a pressing issue in schizophrenia research.

Recently, histamine H3 receptor antagonists (H3R‐ANTs) or inverse agonists (H3R‐IAs) have been proposed as novel treatments for schizophrenia. A radioligand binding study of the brain found remarkable increases in H3 receptor binding in patients with schizophrenia compared with normal controls, indicating that the increased H3 receptors in the prefrontal cortex may be implicated in cognition impairment [8]. H3R‐ANTs/H3R‐IAs increase the synaptic release of histamine and other neurotransmitters, including acetylcholine and glutamate [9], thereby improving synaptoplastic processes linked to memory [10]. Recent animal studies reported that H3R‐ANT‐induced neurotransmitter release leads to postsynaptic receptor pathway activation, such as phosphorylation of cyclic adenosine monophosphate response element binding protein, a transcription factor germane to cognitive function [11]. Thus, H3R‐ANTs/H3R‐IAs may improve cognitive decline in individuals with schizophrenia [10, 11].

To date, 11 double‐blind, randomized, placebo‐controlled trials (DBRPCTs) on H3R‐ANTs/H3R‐IAs (ABT‐288, betahistine, GSK239512, MK‐0249, and pitolisant) for individuals with schizophrenia have been conducted (Table 1) [13, 14, 15, 16, 17, 18, 19, 20, 21, 22]. One DBRPCT showed that betahistine, which is an H1‐receptor agonist and H3R‐ANT [21], improved the Measurement and Treatment Research to Improve Cognition in Schizophrenia Consensus Cognitive Battery (MCCB) composite score and some domain scores of the MCCB [23] compared with placebo [18]; however, other trials failed to demonstrate the superiority of H3R‐ANTs/H3R‐IAs over placebo in the efficacy outcomes for individuals with schizophrenia (Table 1). These discrepant results may be secondary to the small sample sizes of these trials (Table 1) [24]. The meta‐analysis can overcome the limitation of sample size in underpowered studies as a meta‐analysis can increase the statistical power for group comparisons [25]. Hence, a systematic review and meta‐analysis of DBRPCTs of H3R‐ANTs/H3R‐IAs for schizophrenia with respect to the efficacy, acceptability, tolerability, and safety outcomes was conducted.

**TABLE 1:** Study characteristics of the included double‐blind randomized placebo‐controlled trials in our systematic review and meta‐analysis.

(1) Study name, (2) Total *n*, (3) Country, (4) Sponsorship	Study duration (w)	(1) Diagnosis, (2) Status	Intervention (dose, mg/d)	*n*	(1) Age (mean ± SD, y), (2) Female (%), (3) Race/ethnicity (%)	(1) AP (%), (2) Mean AP dose (CHL eq (mg/d)^a^)	SZ symptoms at BL (mean ± SD)	CS of cognitive tests	
								At baseline (mean ± SD)	Result^b^
*Histamine H3 antagonist studies*									
(1) Haig 2014, (2) 214, (3) USA, (4) Industry	12	(1) Stable SZ (DSM‐IV‐TR), (2) OP	ABT‐288 (10)	72	(1) 43.1 ± 9.5, (2) 33.8, (3) White: 37.1	(1) SGAs (100.0), (2) NR	PANSS‐T: 64.4 ± 11.9	MCCB: 28.1 ± 11.9	ABT‐288 = PLA
			ABT‐288 (25)	70					
			PLA	72					
(1) Othman 2014^c^, (2) 67, (3) USA, (4) Industry	2	(1) Stable SZ (DSM‐IV‐TR), (2) OP	ABT‐288 (1)	NR	(1) 42.0 ± 9.0, (2) 22.4, (3) White: 23.9	(1) SGAs (100.0), (2) NR	NR	NR	NR
			ABT‐288 (3)	NR					
			ABT‐288 (6)	NR					
			ABT‐288 (9)	NR					
			ABT‐288 (12)	NR					
			ABT‐288 (15)	NR					
			ABT‐288 (20)	NR					
			ABT‐288 (30)	NR					
			ABT‐288 (45)	NR					
			ABT‐288 (60)	NR					
			PLA	NR					
(1) Jarskog 2015, (2) 50, (3) USA, (4) Industry	7	(1) Stable SZ (DSM‐IV‐TR), (2) OP	GSK239512 (0.08)	25	(1) 39.2 ± 11.2, (2) 21.7, (3) White: 45.7	(1) SGAs and/or FGAs (100.0), (2) NR	BPRS‐T: 29.7 ± 6.6	MCCB: 31.6 ± 12.1	GSK239512 = PLA
			PLA	25					
*Histamine H3 inverse agonist study*									
(1) Egan 2013^d^, (2) 55, (3) Russia and India, (4) Industry	4	(1) Stable SZ (DSM‐IV or DSM‐IV‐TR), (2) OP	MK‐0249 (10)	28	(1) 31.6 ± 7.9, (2) 27.3, (3) White: 63.6	(1) SGAs and/or FGAs (100.0), (2) NR (= < 1000)	PANSS‐T: 56.0 ± 9.1	BACS: 41.7 ± 8.9	MK‐0249 = PLA
			PLA	27					
*Histamine H3 antagonist/inverse agonist study*									
(1) NCT00690274 2019^e^, (2) 52, (3) USA, (4) Academia	12	(1) Stable SZ or SA (DSM‐IV), (2) NR	PIT (20)	23	(1) NR, (2) 76.3, (3) White: 73.7	(1) APs (100.0), (2) NR	NR	NR	NR
			PLA	29					
*BET studies*									
(1) Bai 2023, (2) 94, (3) China, (4) Academia	4	(1) Stable SZ (ICD‐10), (2) IP	BET (48)	47	(1) 46.4 ± 9.6, (2) 39.4, (3) Chinese (100.0)	(1) SGAs (100.0), (2) NR (364.9)	PANSS‐T: 74.7 ± 13.8	NR	NR
			PLA	47					
(1) Barak 2016, (2) 36, (3) Canada and Israel, (4) Industry	16	(1) SZ, SA, SF, or PD‐NOS (DSM‐IV), (2) NR	BET (48)	NR	(1) 26.6 (both adolescents and adults), (2) 28.6, (3) White: 97.1	(1) OLA (100.0), (2) 12.0 (360.0)	NR	NR	NR
			PLA	NR					
(1) Smith 2018a, (2) 40, (3) USA, (4) Academia	12	(1) SZ, SA, SF, or PD‐NOS, ASD, BD (NR), (2) IP or OP	BET (48)	19	(1) 31.1 ± 14.2 (both adolescents and adults), (2) 59.0, (3) White: 33.3	(1) SGAs including CLO and/or FGAs (100.0), (2) NR	BPRS‐T: 32.8 ± 6.7	NR	NR
			PLA	21					
(1) Smith 2018b, (2) 14, (3) China, (4) Academia	12	(1) SZ, SA, SF, or PD‐NOS, ASD, BD (NR), (2) IP or OP	BET (36)	10	(1) NR, (2) NR, (3) NR	(1) SGAs including CLO and/or FGAs (100.0), (2) NR	NR	NR	NR
			PLA	4					
(1) Wang 2021, (2) 89, (3) China, (4) Academia	12	(1) Stable SZ (ICD‐10), (2) IP	BET (72)	45	(1) 47.2 ± 8.9, (2) 39.3, (3) Chinese: 100.0	(1) SGAs (100.0), (2) NR (327.3)	PANSS‐T: 73.2 ± 12.5	MCCB: 39.3 ± 12.2	BET > PLA
			PLA	44					
(1) Poyurovsky 2013, (2) 43, (3) Israel, (4) Academia	6	(1) First‐episode, drug‐naive SZ or SF (DSM‐IV), (2) IP	BET (144) + REB (8)	29	(1) 32.5 ± 9.2, (2) 18.6, (3) NR	(1) OLA (100.0), (2) 10.0 (300.0)	NR	NR	NR
			PLA	14					

Abbreviations: AP, antipsychotic; ASD, autism spectrum disorder; BACS, Brief Assessment of Cognition in Schizophrenia; BD, bipolar disorder; BET, betahistine; BPRS‐T, Brief Psychiatric Rating Scale‐total score; CHL eq, chlorpromazine equivalent; CS, composite score; d, day; DBRPCT, double‐blind randomized placebo‐controlled trial; DSM, Diagnostic and Statistical Manual of Mental Disorders; FGA, first‐generation antipsychotic; ICD, International Classification of Diseases; IP, inpatient; MCCB, Measurement and Treatment Research to Improve Cognition in Schizophrenia Consensus Cognitive Battery; *n*, number of individuals; NR, not report; OLA, olanzapine; OP, outpatient; PANSS‐T, Positive and Negative Syndrome Scale total score; PD‐NOS, psychotic disorder not otherwise specified; PIT, pitolisant; PLA, placebo; REB, reboxetine; SA, schizoaffective disorder; SD, standard deviation; SF, schizophreniform disorder; SGA, second‐generation antipsychotic; SZ, schizophrenia; USA, United States of America; w, weeks; y, years.

^a^ We calculated the CHL eq based on the defined daily doses [12].

^b^ A > B: A was superior to B. A = B: A was similar to B.

^c^ We did not include the Othman [13] study in our meta‐analysis as 21% of the participants were randomized twice.

^d^ Egan [14] study which was a crossover study did not report the first phase results (before crossover).

^e^ The year the study results were registered in ClinicalTrials.gov.

## 2 Methods

This systematic review and meta‐analysis was conducted following the Preferred Reporting Items for Systematic Reviews and Meta‐Analyses statement (Table S1) [26]. This study was registered at the Open Science Framework (https://osf.io/s8ztv).

### 2.1 Search Strategy, Inclusion Criteria, and Data Extraction

Figure S1 summarizes the formal literature search and selection flow of H3R‐ANTs/H3R‐IA trials. At least two authors (Y.N., S.H., and K.S.) simultaneously and independently conducted the literature search and data extraction, and the obtained data were input into a spreadsheet for analysis. All data were evaluated for accuracy by at least two authors (Y.N., S.H., and K.S.). A formal systematic literature review was conducted using the Patient, Intervention, Comparison, and Outcome strategy. Only DBRPCTs of H3R‐ANTs/H3R‐IA were included.

Patient: Individuals with schizophrenia spectrum and other psychotic disorders who received antipsychotics.

Intervention: H3R‐ANT/H3R‐IAs (i.e., A‐349821, ABT‐239, ABT‐288, ABT‐652, APD‐916, AZD5213, betahistine, BF2.649, burimamide, CEP‐26401, ciproxifan, clobenpropit, conessine, GSK‐1004723, GSK‐189254, GSK‐239512, GSK‐835726, GW784568X, impentamine, iodophenpropit, irdabisant, JNJ‐17216498, JNJ‐31001074, JNJ‐39220675, JNJ‐5207852, LML‐134, MK‐0249, MK‐3134, MK‐7288, PF‐03654746, pitolisant, S 38093, SAR‐110894, SCH‐497079, SUVN‐G3031, thioperamide, triprolisant, and VUF5681) that were introduced as a H3R‐ANT/H3R‐IA in the following previous reviews [27, 28, 29].

Comparison: Placebo.

Outcomes: See the following section.

In the databases of Embase, PubMed, and the Cochrane Central Register of Controlled Trials, the authors searched for trials published before December 7, 2024. The detailed information regarding our literature search method was demonstrated in Figure S1. The retrieved trials were assessed against the inclusion and exclusion criteria, and those eligible were selected. In the reference lists of the included trials and review articles, additional relevant published and unpublished trials, including conference abstracts, were manually searched. Clinical trial registries (e.g., ClinicalTrials.gov [http://clinicaltrials.gov/] and the World Health Organization International Clinical Trials Registry Platform [http://www.who.int/ictrp/search/en/]) were also searched to ascertain that the eligible trials were comprehensive and to minimize the influence of publication bias. A consensus was achieved among the authors to resolve any discrepancies in the selected trials (Y.N., S.H., K.S., and T.K.).

### 2.2 Outcome Measures and Data Synthesis

The primary outcome was the cognitive function scale's composite score based on the MCCB and Brief Assessment of Cognition in Schizophrenia (BACS) [30] (Table S2). Other outcomes included seven domain scores of cognitive function (speed of processing [only MCCB], attention/vigilance [only MCCB], working memory [MCCB and BACS + University of Pennsylvania Computerized Neuropsychological battery [31]], verbal learning [only MCCB], visual learning [MCCB and Brief Visuospatial Memory Test‐Revised [32]], reasoning/problem solving [only MCCB], and social cognition [only MCCB]), University of California San Diego Performance‐Based Skills Assessment (UPSA) [33] score, schizophrenia total symptom score based on the total scores for the Positive and Negative Syndrome Scale (PANSS) [34] and the Brief Psychiatric Rating Scale [35], positive symptom scores based on the scores for PANSS positive subscale and Scale for the Assessment of Positive Symptoms [36], negative symptom scores based on the scores for PANSS negative subscale, Scale for the Assessment of Negative Symptoms [37] and 16‐item Negative Symptom Assessment Scale [38], PANSS general subscale score, depressive symptom scale score based on the scores for Hamilton Depression Rating Scale [39] and Montgomery Åsberg Depression Rating Scale [40], Clinical Global Impressions‐Severity [41] score, all‐cause discontinuation rate, discontinuation rate due to adverse event, and incidence of individual adverse events (continuous variables: body mass index, body weight, waist circumference, hip circumference, serum triglyceride levels, low‐density lipoprotein cholesterol levels, and total cholesterol levels; dichotomous variables: incidence of anxiety, anorexia/decreased appetite/loss of appetite, insomnia, middle insomnia, abnormal dream, dizziness, tremor, headache, nausea, nasopharyngitis, diarrhea, constipation, increased serum antipsychotic, alanine aminotransferase, aspartate aminotransferase, eosinophil levels, and decreased serum neutrophil levels). If at least two studies in each drug group have sufficient data to conduct a meta‐analysis for a specific outcome, a meta‐analysis was conducted for those outcomes. For the efficacy outcome, we also assessed the statistical results derived from only one study.

Our meta‐analysis primarily used data based on the intention‐to‐treat or full analysis set principles. If required data were missing from the studies, we attempted to contact the original investigators to obtain unpublished data. However, we did not obtain the any unpublished data.

## 3 Meta‐Analysis Methods

ABT‐288, betahistine, betahistine + reboxetine, GSK239512, MK‐0249, and pitolisant were evaluated by our systematic review and meta‐analysis. Only betahistine has an H1‐receptor agonistic reaction and H3‐receptor antagonistic reaction [21], although other drugs only have H3‐receptor antagonistic/inverse agonistic reaction. Thus, as a difference in the receptor pharmacology between betahistine and others exists, we conducted a meta‐analysis for all outcomes divided by betahistine or other pooled H3R‐ANTs/H3R‐IAs. A random‐effects pairwise meta‐analysis was conducted based on the aforementioned outcomes to compare betahistine or other pooled H3R‐ANTs/H3R‐IAs with placebo [42]. The estimated standardized mean differences (SMD) in the continuous data and risk ratios (RR) for dichotomous data were calculated, with their respective 95% confidence intervals (CIs). The heterogeneity of the included trials was evaluated using the *I^2^* statistic, with *I* ^2^ ≥ 50% as considerable heterogeneity [24]. As lower scores (e.g., cognitive function scales and UPSA scores) indicate a higher impairment or symptom severity, the algebraic sign of the numerical scores for these scales was reversed. The risk of bias was evaluated using the Cochrane risk‐of‐bias tool for randomized trials version 2 [24]. To determine potential publication bias as funnel plots with < 10 studies were not meaningful, Egger's test was performed [24]. Statistical analysis of our meta‐analysis was performed using Comprehensive Meta‐Analysis, version 4 (Biostat Inc., Englewood, NJ, USA).

## 4 Results

### 4.1 Study Characteristics

A total of 73 articles were retrieved during the initial search, of which 24 were discarded as duplicates. A review of the abstract and/or title of the remaining articles and trials ruled out 39 studies (Figure S1). The clinical trial registries and the manual search did not reveal further trials. Finally, in our systematic review and meta‐analysis, 11 trials involving a total of 754 individuals were included (Figure S1). A study by Smith et al. (2018) included two trials [22]. Two trials included ABT‐288 [13, 16], one trial included GSK239512 [17], one trial included MK‐0249 [14], one trial included pitolisant [19], five trials included betahistine [15, 18, 20, 22], and one trial included combination therapy of betahistine with reboxetine (betahistine+reboxetine) [21]. In Table 1, the study characteristics are illustrated. Two trials included adolescents and adults [20, 22]. One trial only included individuals with first‐episode, drug‐naive schizophrenia and schizophreniform disorder [21]. Five trials were conducted in the USA [13, 16, 17, 19, 22], and three trials were conducted in China [15, 18, 22]. Although all trials included individuals with schizophrenia who received antipsychotics, six trials only included individuals who received second‐generation antipsychotics [13, 15, 16, 18, 20, 21]. Six trials were not industry‐sponsored [15, 18, 19, 21, 22]. Most of the trials included were evaluated as having high risk or some concerns regarding the overall risk of bias (Figure S2). As the Egan [14] study, which was a crossover study, did not report the first phase results (before crossover), this study was evaluated as having a high risk of bias in domain 2 (due to inappropriate analysis). As 21% of the participants in the Othman [13] study were participants in the same study twice, the study was evaluated as having a high risk of bias in domain 2 (due to inappropriate analysis). In our meta‐analysis, we did not include data from the Othman [13] study because there was no available data for the meta‐analysis.

### 4.2 Systematic Review and Meta‐Analysis Results

**FIGURE 1:**
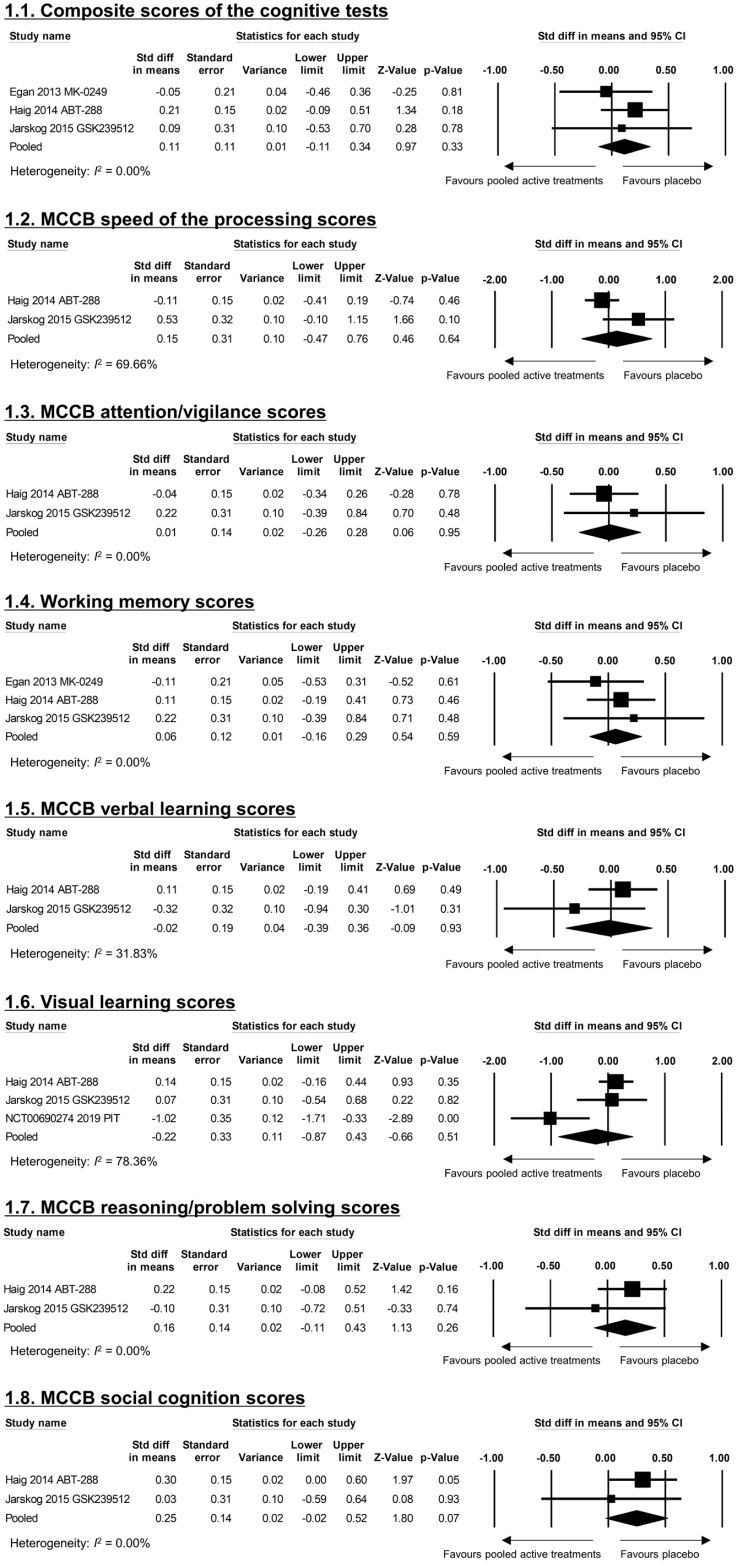
Forest plot of cognitive function for pooled H3R‐ANTs/H3R‐IAs other than betahistine. (a) Composite scores of the cognitive tests. (b) MCCB speed of the processing scores. (c) MCCB attention/vigilance scores. (d) Working memory scores. (e) MCCB verbal learning scores. (f) Visual learning scores. (g) MCCB reasoning/problem solving scores. (h) MCCB social cognition scores. 95% CI, 95% confidence interval; MCCB, Measurement and Treatment Research to Improve Cognition in Schizophrenia Consensus Cognitive Battery; PIT, pitolisant; Std diff in means, standardized difference in means.

Betahistine outperformed placebo in terms of the improvement of overall cognitive symptoms (SMD [95% CI] = −0.61 (−1.03, −0.18)), speed of processing (−0.44 [−0.87, −0.02]), attention/vigilance (−0.43 [−0.85, −0.01]), working memory (−0.48 [−0.90, −0.06]), verbal learning (−0.62 [−1.04, −0.19]), and visual learning (−0.57 [−1.00, −0.15]) (Table 2). Betahistine + reboxetine outperformed placebo regarding the improvement of depressive symptoms (−4.04 [−5.10, −2.97]) (Table 2). However, the results were derived from one betahistine study [18] and betahistine+reboxetine [21]. Another study reported that pitolisant outperformed placebo regarding the improvement of depressive symptoms (SMD [95% CI] = −3.24 [−4.22, −2.26]) [19] (Table 2). One other study reported that ABT‐288 was inferior to placebo regarding the CGI‐S score (SMD [95% CI] = 0.31 [0.02, 0.61]) [16] (Table 2). Other pooled H3R‐ANTs/H3R‐IAs did not outperform placebo regarding any efficacy outcomes (Figure 1 and Table 2).

**TABLE 2:** The efficacy outcome results.

Outcome	Drug^a^	*K*	*n*	SMD	95% CI	*p* ^b^	Heterogeneity
Composite scores of the cognitive tests	BET	1	89	−0.61	−1.03, −0.18	**0.01**	na
	Others	3	322	0.11	−0.11, 0.34	0.33	*I* ^2^ = 0.00%
MCCB speed of the processing scores	BET	1	89	−0.44	−0.87, −0.02	**0.04**	na
	Others	2	229	0.15	−0.47, 0.76	0.64	*I* ^2^ = 69.66%
MCCB attention/vigilance scores	BET	1	89	−0.43	−0.85, −0.01	**0.04**	na
	Others	2	229	0.01	−0.26, 0.28	0.95	*I* ^2^ = 0.00%
Working memory scores	BET	1	89	−0.48	−0.90, −0.06	**0.02**	na
	Others	3	316	0.06	−0.16, 0.29	0.59	*I* ^2^ = 0.00%
MCCB verbal learning scores	BET	1	89	−0.62	−1.04, −0.19	**0.00** *	na
	Others	2	229	−0.02	−0.39, 0.36	0.93	*I* ^2^ = 31.83%
Visual learning scores	BET	1	89	−0.57	−1.00, −0.15	**0.01**	na
	Others	3	267	−0.22	−0.87, 0.43	0.51	*I* ^2^ = 78.36%
MCCB reasoning/problem solving scores	BET	1	89	−0.41	−0.83, 0.01	0.06	na
	Others	2	229	0.16	−0.11, 0.43	0.26	*I* ^2^ = 0.00%
MCCB social cognition scores	BET	1	89	−0.28	−0.69, 0.14	0.20	na
	Others	2	229	0.25	−0.02, 0.52	0.07	*I* ^2^ = 0.00%
UPSA scores	Others	2	207	0.12	−0.18, 0.41	0.45	*I* ^2^ = 5.24%
Total symptom scores	BET	4	249	−0.11	−0.36, 0.14	0.38	*I* ^2^ = 0.00%
	Others	3	324	0.09	−0.16, 0.34	0.50	*I* ^2^ = 14.58%
Positive symptom scores	BET	3	226	−0.22	−0.48, 0.05	0.11	*I* ^2^ = 0.00%
	Other	1	94	0.13	−0.28, 0.53	0.53	na
Negative symptom scores	BET	3	226	−0.11	−0.38, 0.15	0.39	*I* ^2^ = 0.00%
	Others	3	334	0.02	−0.22, 0.26	0.85	*I* ^2^ = 11.06%
PANSS general subscale scores	BET	2	183	0.12	−0.17, 0.41	0.42	*I* ^2^ = 0.00%
Depressive symptom scores	BET^c^	1	43	−4.04	−5.10, −2.97	**0.00** **	na
	Other^d^	1	38	−3.24	−4.22, −2.26	**0.00** **	na
CGI‐S scores	BET^c^	1	43	−0.20	−0.84, 0.44	0.55	na
	Other^e^	1	198	0.31	0.02, 0.61	**0.04**	na

Abbreviations: 95% CI, 95% confidence interval; BET, betahistine; CGI‐S, Clinical Global Impressions‐Severity; K, number of studies; MCCB, Measurement and Treatment Research to Improve Cognition in Schizophrenia Consensus Cognitive Battery; *n*, number of individuals; na, not applicable; PANSS, Positive and Negative Syndrome Scale; SMD, standardized mean difference; UPSA, University of California and San Diego Performance Based Skills Assessment.

^a^ Others: pooled histamine H3 receptor antagonist or inverse agonist other than betahistine.

^b^ Boldface indicates statistical significance.

^c^ Betahistine+reboxetine study.

^d^ Pitolisant study.

^e^ ABT‐288 study.

* 0.004.

** < 0.000000001.

Other pooled H3R‐ANTs/H3R‐IAs had a risk of insomnia (RR [95% CI] = 2.18 [1.05, 4.55]) (Table 3). However, no differences were found in terms of acceptability, tolerability, and other safety outcomes between betahistine or other pooled H3R‐ANTs/H3R‐IAs and placebo (Table 3).

**TABLE 3:** The results of the acceptability, tolerability, and safety outcomes.

Continuous variable							
Outcome	Drug	*K*	*n*	SMD	95% CI	*p*	Heterogeneity
Body mass index	BET	3	176	−0.33	−1.00, 0.34	0.34	*I* ^2^ = 76.275
Body weight	BET	3	176	−0.28	−0.96, 0.39	0.41	*I* ^2^ = 76.66%
Waist circumference	BET	2	133	0.13	−0.21, 0.47	0.45	*I* ^2^ = 0.00%
Hip circumference	BET	2	133	0.18	−0.16, 0.52	0.31	*I* ^2^ = 0.00%
Serum triglyceride levels	BET	2	122	−0.15	−0.51, 0.20	0.41	*I* ^2^ = 0.00%
Serum low‐density lipoprotein cholesterol levels	BET	2	122	−0.09	−0.45, 0.26	0.60	*I* ^2^ = 0.00%
Serum total cholesterol levels	BET	2	122	0.01	−0.34, 0.37	0.95	*I* ^2^ = 0.00%

Dichotomous variable							
Outcome	Drug^a^	*K*	*n*	RR	95% CI	*p* ^b^	Heterogeneity
All‐cause discontinuation	BET	5	280	0.84	0.45, 1.58	0.59	*I* ^2^ = 21.25%
	Others	4	371	1.54	0.97, 2.43	0.07	*I* ^2^ = 0.00%
Discontinuation due to adverse events	Others	3	318	3.50	0.98, 12.53	0.05	*I* ^2^ = 0.00%
Anxiety	Others	2	316	3.22	0.80, 12.95	0.10	*I* ^2^ = 0.00%
Anorexia/decreased appetite/loss of appetite	BET	2	128	1.23	0.48, 3.15	0.67	*I* ^2^ = 0.00%
Insomnia	Others	3	366	2.18	1.05, 4.55	**0.04**	*I* ^2^ = 0.00%
Middle insomnia	Others	2	153	2.00	0.45, 8.79	0.36	*I* ^2^ = 0.00%
Abnormal dream	Others	2	263	1.78	0.44, 7.15	0.42	*I* ^2^ = 0.00%
Dizziness	Others	2	263	2.92	0.77, 11.09	0.12	*I* ^2^ = 0.00%
Tremor	BET	3	164	0.94	0.36, 2.46	0.89	*I* ^2^ = 11.31%
Headache	Others	3	365	1.68	0.79, 3.58	0.18	*I* ^2^ = 0.00%
Nausea	BET	2	128	1.01	0.31, 3.31	0.99	*I* ^2^ = 0.00%
	Others	3	366	1.35	0.40, 4.58	0.63	*I* ^2^ = 31.88%
Nasopharyngitis	Others	2	316	1.08	0.29, 4.01	0.91	*I* ^2^ = 0.00%
Diarrhea	Others	2	263	1.48	0.39, 5.57	0.57	*I* ^2^ = 0.00%
Constipation	BET	2	128	0.45	0.13, 1.57	0.21	*I* ^2^ = 58.13%
Increased blood levels of antipsychotics	BET	2	183	2.97	0.31, 28.01	0.34	*I* ^2^ = 0.00%
Increased blood levels of ALT	Others	2	147	1.32	0.19, 9.36	0.78	*I* ^2^ = 32.29%
Increased blood levels of AST	Others	2	147	0.50	0.07, 3.78	0.51	*I* ^2^ = 0.00%
Increased blood levels of eosinophils	Others	2	147	0.34	0.04, 3.19	0.35	*I* ^2^ = 0.00%
Decreased blood levels of neutrophils	Others	2	147	0.34	0.04, 3.19	0.35	*I* ^2^ = 0.00%

Abbreviations: 95% CI, 95% confidence interval; ALT, alanine aminotransferase; AST, aspartate aminotransferase; BET, betahistine; *K*, number of studies; LDL, low‐density lipoprotein; *n*, number of individuals; RR, risk ratio; SMD, standardized mean difference.

^a^ Others: histamine H3 receptor antagonist or inverse agonist other than betahistine.

^b^ Boldface indicates statistical significance.

## 5 Discussion

Our systematic review and meta‐analysis for individuals afflicted with schizophrenia showed that betahistine improves cognitive symptoms, and betahistine + reboxetine and pitolisant enhance depressive symptoms. Thus, we anticipate that these drugs may become novel candidate drugs for individuals with schizophrenia. However, each result was derived from only one trial [18, 19, 21]. Therefore, conducting another study to replicate the findings will be required to find robust evidence.

Pooled H3R‐ANTs/H3R‐IAs (MK‐0249, ABT‐288, and GSK239512) had a risk of insomnia. However, no differences were found in terms of acceptability, tolerability, and other safety outcomes between betahistine or other pooled H3R‐ANTs/H3R‐IAs and placebo. No studies of betahistine, betahistine + reboxetine, or pitolisant reported the incidence of insomnia. Thus, H3R‐ANTs/H3R‐IAs might have good overall acceptability and tolerability for schizophrenia.

Recent network meta‐analysis revealed that any antipsychotics failed to improve cognitive symptoms in people with schizophrenia [43]. Betahistine has an H1‐receptor agonistic and H3‐receptor antagonistic reaction and is indicated for the treatment of Meniere's disease, symptoms of which may include vertigo. H3‐receptor antagonistic reaction of betahistine increase the synaptic release of histamine and other neurotransmitters, including acetylcholine and glutamate [9], thereby improving synaptoplastic processes linked to cognitive function [10]. However, other pooled H3R‐ANTs/H3R‐IAs did not outperform placebo regarding any efficacy outcomes. Histaminergic H1 agonists promote wakefulness [44]. As several antipsychotics such as quetiapine, olanzapine, and clozapine have H1‐receptor antagonistic reaction, betahistine might improve cognitive symptoms in schizophrenia through reduced sedative effects associated with antipsychotics [3, 45]. Additionally, most antipsychotics do not act on H3 receptors.

Our systematic review found that betahistine+reboxetine and pitolisant improved depressive symptoms in people with schizophrenia. Since reboxetine is an antidepressant [46], its antidepressant effect could influence the result. No differences were found in the results of all outcomes between a subgroup including only betahistine studies and another subgroup including betahistine studies and betahistine +reboxetine study (Table S3). Pitolisant is a histamine‐3 receptor (H3R), a competitive antagonist and inverse agonist, acting through the histamine system to regulate wakefulness [47, 48]. Other neurotransmitters are also modulated by pitolisant, increasing acetylcholine, noradrenaline, and dopamine release in the brain. The effects could enhance the depressive symptoms in schizophrenia [48].

ABT‐288 was inferior to placebo in terms of the CGI‐S score [16]. However, the result was derived from only one study. Moreover, as the appropriate dose of ABT‐288 for schizophrenia was unknown, the interpretation of the results should be exercised with caution.

However, our study had several limitations. First, the positive results were derived from only one study. Another limitation was the short study duration of each trial (2–16 weeks). A longer‐duration study using a larger sample is required as individuals with schizophrenia have a long‐term treatment period. Third, we did not explore which antipsychotics were compatible with betahistine or H3R‐ANTs/H3R‐IAs.

In conclusion, although our systematic review and meta‐analysis suggested that some H3R‐ANTs/H3R‐IAs appeared to have benefits for the treatment of cognitive symptoms and depressive symptoms in individuals with schizophrenia, the results were derived from only one trial. A replication randomized trial of the drugs should be conducted using a larger sample size.

## Author Contributions

Dr. Yasufumi Nishii and Dr. Kenji Sakuma had complete access to all study data and is responsible for data integrity and analysis accuracy. Prof. Taro Kishi assisted in the conceptualization and design of the study. Dr. Yasufumi Nishii, Dr. Kenji Sakuma, and Prof. Taro Kishi performed the statistical analysis. The acquisition and interpretation of data were handled by Dr. Yasufumi Nishii, Dr. Kenji Sakuma, and Dr. Shun Hamanaka. The manuscript was written by all of the authors. The review was supervised by Prof. Nakao Iwata.

## Ethics Statement

The authors have nothing to report.

## Consent

The authors have nothing to report.

## Conflicts of Interest

The authors declare no conflicts of interest related to the subject of this study. Interests for the past 3 years are as follows: Dr. Yasufumi Nishii has received speaker's honoraria from Sumitomo. Dr. Kenji Sakuma has received speaker's honoraria from Daiichi Sankyo, Janssen, Kyowa, Meiji, Mitsubishi‐Tanabe, Otsuka, Sumitomo, and Takeda and has received grants from JSPS KAKENHI (19K17099 and 23K06998), and Japan Agency for Medical Research and Development (JP22dk0307107, JP23dk0307122, JP24dk0307122, and JP25dk0307122). Dr. Shun Hamanaka has received speaker's honoraria from Sumitomo. Prof. Nakao Iwata received speaker's honoraria from Sumitomo, Eisai, Janssen, Otsuka, Meiji, Shionogi, Takeda, Yoshitomiyakuhin, and Viatris and research grants from Eisai, Takeda, Sumitomo, and Otsuka. Prof. Taro Kishi has received speaker's honoraria from Eisai, Janssen, Meiji, Otsuka, Sumitomo, Takeda, Mitsubishi‐Tanabe, Kyowa, Boehringer Ingelheim, and Viatris and research grants from Eisai, JSPS KAKENHI (19K08082 and 23K06998), Japan Agency for Medical Research and Development (JP22dk0307107, JP22wm0525024, 23dk0307117h0001, and 24dk0307129h0001), and the Japanese Ministry of Health, Labour and Welfare (21GC1018).

## Supporting information


Data S1.

## Data Availability

Data used for the current study were reported in articles as cited in this paper.
