# Supplementary material for: Efficacy and Safety of Histamine H3 Receptor Antagonist/Inverse Agonist Including Betahistine for Schizophrenia: A Systematic Review and Meta‐Analysis
Source: Neuropsychopharmacol Rep. 2025 Jun 26;45(3):e70034. doi: 10.1002/npr2.70034 (PMC12198695; doi:10.1002/npr2.70034)
Supplement: Supplementary file 1 — Data S1. [file NPR2-45-e70034-s001.docx]

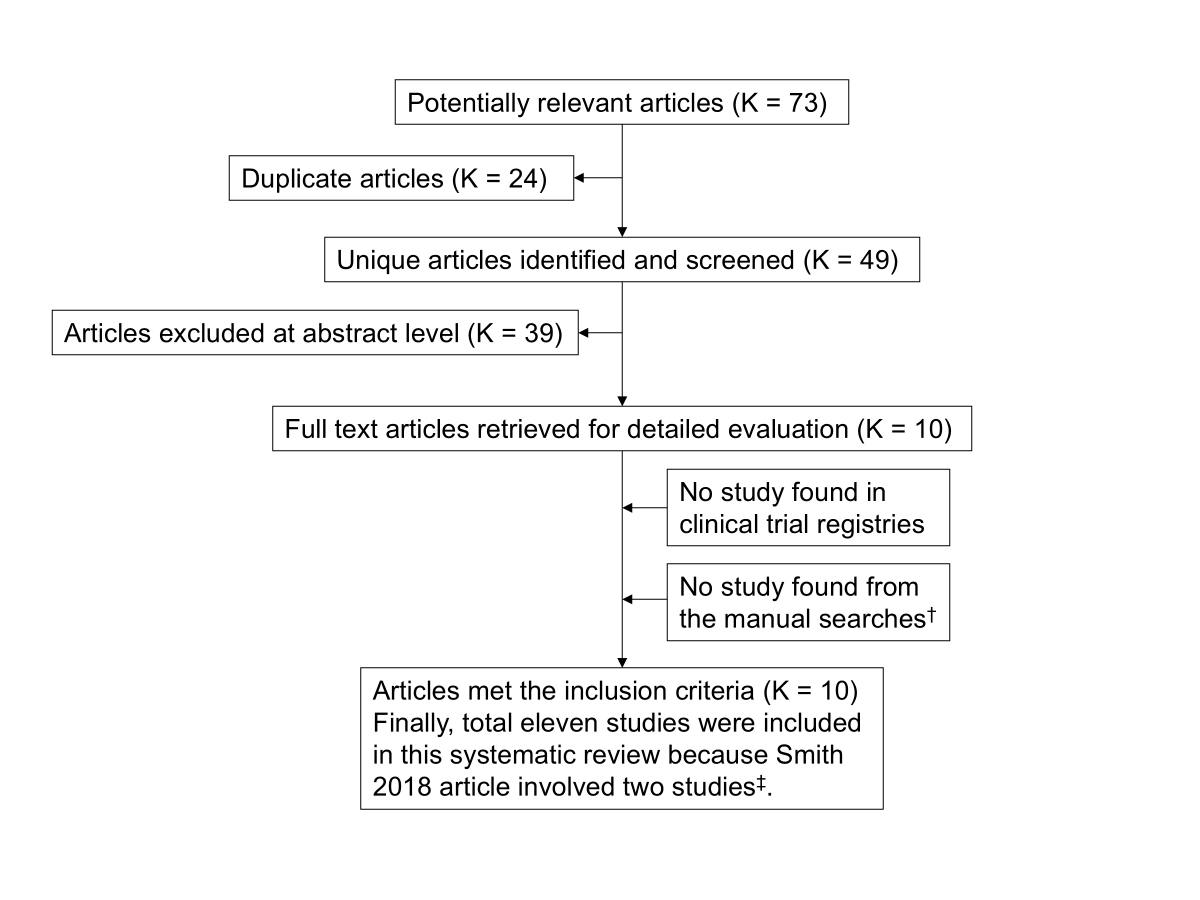
**Figure S1. PRISMA flow diagram**

The search terms in PubMed and The Cochrane Library databases included ("histamine H3 receptor antagonist*" OR "H3 receptor antagonist*" OR "histamine H3 receptor inverse agonist*" OR "H3 receptor inverse agonist*" OR A-349821 OR ABT-239 OR ABT-288 OR ABT-652 OR APD-916 OR AZD5213 OR betahistine OR BF2.649 OR burimamide OR CEP-26401 OR ciproxifan OR clobenpropit OR conessine OR GSK-1004723 OR GSK-189254 OR GSK-239512 OR GSK-835726 OR GW784568X OR impentamine OR iodophenpropit OR irdabisant OR JNJ-17216498 OR JNJ-31001074 OR JNJ-39220675 OR JNJ-5207852 OR LML-134 OR MK-0249 OR MK-3134 OR MK-7288 OR PF-03654746 OR pitolisant OR "S 38093" OR SAR-110894 OR SCH-497079 OR SUVN-G3031 OR thioperamide OR triprolisant OR VUF5681) AND (schizophrenia) AND (random*). The search terms for Embase included ('histamine H3 receptor antagonist*' OR 'H3 receptor antagonist*' OR 'histamine H3 receptor inverse agonist*' OR 'H3 receptor inverse agonist*' OR A-349821 OR ABT-239 OR ABT-288 OR ABT-652 OR APD-916 OR AZD5213 OR betahistine OR BF2.649 OR burimamide OR CEP-26401 OR ciproxifan OR clobenpropit OR conessine OR GSK-1004723 OR GSK-189254 OR GSK-239512 OR GSK-835726 OR GW784568X OR impentamine OR iodophenpropit OR irdabisant OR JNJ-17216498 OR JNJ-31001074 OR JNJ-39220675 OR JNJ-5207852 OR LML-134 OR MK-0249 OR MK-3134 OR MK-7288 OR PF-03654746 OR pitolisant OR 'S 38093' OR SAR-110894 OR SCH-497079 OR SUVN-G3031 OR thioperamide OR triprolisant OR VUF5681) AND (schizophrenia/exp OR schizophrenia) AND (random*).

† Agarwal SM, Stogios N, Ahsan ZA, Lockwood JT, Duncan MJ, Takeuchi H, Cohn T, Taylor VH, Remington G, Faulkner GEJ, Hahn M. Pharmacological interventions for prevention of weight gain in people with schizophrenia. Cochrane Database Syst Rev. 2022 Oct 3;10(10):CD013337.

‡ Smith RC, Maayan L, Wu R, Youssef M, Jing Z, Sershen H, Szabo V, Meyers J, Jin H, Zhao J, Davis JM. Betahistine effects on weight-related measures in patients treated with antipsychotic medications: a double-blind placebo-controlled study. Psychopharmacology (Berl). 2018 Dec;235(12):3545-3558.

**Figure S2. Risk of bias summary**

|  | Domain 1 | Domain 2 | Domain 3 | Domain 4 | Domain 5 | Overall risk of bias |
| --- | --- | --- | --- | --- | --- | --- |
|  | Randomization process | Deviation from intended intervention | Missing outcome data | Measurement of the outcome | Selection of the reported result |  |
| Bai 2023 | Low risk | Low risk | Some concerns | Low risk | Some concerns | Some concerns |
| Barak 2016 | Low risk | Low risk | High risk | Low risk | Some concerns | High risk |
| Egan 2013 | Low risk | High risk^†^ | Some concerns | Low risk | Low risk | High risk |
| Haig 2014 | Low risk | Low risk | Low risk | Low risk | Some concerns | Some concerns |
| Jarskog 2015 | Low risk | Low risk | Low risk | Low risk | Low risk | Low risk |
| Othman 2014 | Low risk | High risk^‡^ | High risk | Low risk | Some concerns | High risk |
| Poyurovsky 2013 | Some concerns | Low risk | Some concerns | Some concerns | Some concerns | Some concerns |
| Smith 2018a | Low risk | Low risk | Low risk | Low risk | Some concerns | Some concerns |
| Smith 2018b | Some concerns | High risk | Low risk | High risk | Some concerns | High risk |
| Wang 2021 | Low risk | Low risk | Some concerns | Low risk | Some concerns | Some concerns |
| NCT00690274 2019 | Some concerns | High risk | Some concerns | Some concerns | Low risk | High risk |

Version 2 of the Cochrane risk-of-bias tool for randomized trials (<https://www.riskofbias.info/>)

† Egan 2013 study which was a crossover study did not report the results in the first phase (before crossover). Therefore, this study was assessed as having a high risk of bias in domain 2 (due to inappropriate analysis).

‡ Othman 2014 study was assessed as having a high risk of bias in domain 2 (due to inappropriate analysis) because 21% of the participants were randomized twice.

**Table S1. PRISMA checklist.**

| **Section and Topic** | **Item #** | **Checklist item** | **Location where item is reported** |
| --- | --- | --- | --- |
| **TITLE** | | |  |
| Title | 1 | Identify the report as a systematic review. | P1 |
| **ABSTRACT** | | |  |
| Abstract | 2 | See the PRISMA 2020 for Abstracts checklist. | P2 |
| **INTRODUCTION** | | |  |
| Rationale | 3 | Describe the rationale for the review in the context of existing knowledge. | P3 |
| Objectives | 4 | Provide an explicit statement of the objective(s) or question(s) the review addresses. | P3 |
| **METHODS** | | |  |
| Eligibility criteria | 5 | Specify the inclusion and exclusion criteria for the review and how studies were grouped for the syntheses. | P3-5 |
| Information sources | 6 | Specify all databases, registers, websites, organisations, reference lists and other sources searched or consulted to identify studies. Specify the date when each source was last searched or consulted. | P3-5 |
| Search strategy | 7 | Present the full search strategies for all databases, registers and websites, including any filters and limits used. | P3-5 |
| Selection process | 8 | Specify the methods used to decide whether a study met the inclusion criteria of the review, including how many reviewers screened each record and each report retrieved, whether they worked independently, and if applicable, details of automation tools used in the process. | P3-5 |
| Data collection process | 9 | Specify the methods used to collect data from reports, including how many reviewers collected data from each report, whether they worked independently, any processes for obtaining or confirming data from study investigators, and if applicable, details of automation tools used in the process. | P3-5 |
| Data items | 10a | List and define all outcomes for which data were sought. Specify whether all results that were compatible with each outcome domain in each study were sought (e.g. for all measures, time points, analyses), and if not, the methods used to decide which results to collect. | P3-5 |
|  | 10b | List and define all other variables for which data were sought (e.g. participant and intervention characteristics, funding sources). Describe any assumptions made about any missing or unclear information. | P3-5 |
| Study risk of bias assessment | 11 | Specify the methods used to assess risk of bias in the included studies, including details of the tool(s) used, how many reviewers assessed each study and whether they worked independently, and if applicable, details of automation tools used in the process. | P3-5 |
| Effect measures | 12 | Specify for each outcome the effect measure(s) (e.g. risk ratio, mean difference) used in the synthesis or presentation of results. | P3-5 |
| Synthesis methods | 13a | Describe the processes used to decide which studies were eligible for each synthesis (e.g. tabulating the study intervention characteristics and comparing against the planned groups for each synthesis (item #5)). | P3-5 |
|  | 13b | Describe any methods required to prepare the data for presentation or synthesis, such as handling of missing summary statistics, or data conversions. | P3-5 |
|  | 13c | Describe any methods used to tabulate or visually display results of individual studies and syntheses. | P3-5 |
|  | 13d | Describe any methods used to synthesize results and provide a rationale for the choice(s). If meta-analysis was performed, describe the model(s), method(s) to identify the presence and extent of statistical heterogeneity, and software package(s) used. | P3-5 |
|  | 13e | Describe any methods used to explore possible causes of heterogeneity among study results (e.g. subgroup analysis, meta-regression). | P3-5 |
|  | 13f | Describe any sensitivity analyses conducted to assess robustness of the synthesized results. | P3-5 |
| Reporting bias assessment | 14 | Describe any methods used to assess risk of bias due to missing results in a synthesis (arising from reporting biases). | P3-5 |
| Certainty assessment | 15 | Describe any methods used to assess certainty (or confidence) in the body of evidence for an outcome. | P3-5 |
| **RESULTS** | | |  |
| Study selection | 16a | Describe the results of the search and selection process, from the number of records identified in the search to the number of studies included in the review, ideally using a flow diagram. | P5 |
|  | 16b | Cite studies that might appear to meet the inclusion criteria, but which were excluded, and explain why they were excluded. | P5 |
| Study characteristics | 17 | Cite each included study and present its characteristics. | P5 |
| Risk of bias in studies | 18 | Present assessments of risk of bias for each included study. | P5 |
| Results of individual studies | 19 | For all outcomes, present, for each study: (a) summary statistics for each group (where appropriate) and (b) an effect estimate and its precision (e.g. confidence/credible interval), ideally using structured tables or plots. | P5 |
| Results of syntheses | 20a | For each synthesis, briefly summarise the characteristics and risk of bias among contributing studies. | P5 |
|  | 20b | Present results of all statistical syntheses conducted. If meta-analysis was done, present for each the summary estimate and its precision (e.g. confidence/credible interval) and measures of statistical heterogeneity. If comparing groups, describe the direction of the effect. | P5 |
|  | 20c | Present results of all investigations of possible causes of heterogeneity among study results. | P5 |
|  | 20d | Present results of all sensitivity analyses conducted to assess the robustness of the synthesized results. | P5 |
| Reporting biases | 21 | Present assessments of risk of bias due to missing results (arising from reporting biases) for each synthesis assessed. | P5 |
| Certainty of evidence | 22 | Present assessments of certainty (or confidence) in the body of evidence for each outcome assessed. | P5 |
| **DISCUSSION** | | |  |
| Discussion | 23a | Provide a general interpretation of the results in the context of other evidence. | P6 |
|  | 23b | Discuss any limitations of the evidence included in the review. | P6 |
|  | 23c | Discuss any limitations of the review processes used. | P6 |
|  | 23d | Discuss implications of the results for practice, policy, and future research. | P6 |
| **OTHER INFORMATION** | | |  |
| Registration and protocol | 24a | Provide registration information for the review, including register name and registration number, or state that the review was not registered. | P3 |
|  | 24b | Indicate where the review protocol can be accessed, or state that a protocol was not prepared. | P3 |
|  | 24c | Describe and explain any amendments to information provided at registration or in the protocol. | P3 |
| Support | 25 | Describe sources of financial or non-financial support for the review, and the role of the funders or sponsors in the review. | P7 |
| Competing interests | 26 | Declare any competing interests of review authors. | P7 |
| Availability of data, code and other materials | 27 | Report which of the following are publicly available and where they can be found: template data collection forms; data extracted from included studies; data used for all analyses; analytic code; any other materials used in the review. | Supplementary material |

*From:*  Page MJ, McKenzie JE, Bossuyt PM, Boutron I, Hoffmann TC, Mulrow CD, et al. The PRISMA 2020 statement: an updated guideline for reporting systematic reviews. BMJ 2021;372:n71. doi: 10.1136/bmj.n71

For more information, visit: <http://www.prisma-statement.org/>

**Table S2. Data synthesis for efficacy outcomes**

| **Cognitive tests** | **Composite scores** | **Speed of processing** | **Attention/**  **vigilance** | **Working memory** | **Verbal learning** | **Visual learning** | **Reasoning/**  **problem solving** | **Social cognition** |
| --- | --- | --- | --- | --- | --- | --- | --- | --- |
| Bai 2023 |  |  |  |  |  |  |  |  |
| Barak 2016 |  |  |  |  |  |  |  |  |
| Egan 2013 | BACS |  |  | BACS + CNB^†^ |  |  |  |  |
| Haig 2014 | MCCB | MCCB | MCCB | MCCB | MCCB | MCCB | MCCB | MCCB |
| Jarskog 2015 | MCCB | MCCB | MCCB | MCCB | MCCB | MCCB | MCCB | MCCB |
| Othman 2014 |  |  |  |  |  |  |  |  |
| Poyurovsky 2013 |  |  |  |  |  |  |  |  |
| Smith 2018a |  |  |  |  |  |  |  |  |
| Smith 2018b |  |  |  |  |  |  |  |  |
| Wang 2021 | MCCB | MCCB | MCCB | MCCB | MCCB | MCCB | MCCB | MCCB |
| NCT00690274 2019 |  |  |  |  |  | BVMT-R |  |  |

| **Psychopathology** | **Total symptom** | **Positive symptom** | **Negative symptom** | **Depressive symptom** |
| --- | --- | --- | --- | --- |
| Bai 2023 | PANSS-T | PANSS-P | PANSS-N |  |
| Barak 2016 | PANSS-T |  |  |  |
| Egan 2013 | PANSS-T | PANSS-P | PANSS-N |  |
| Haig 2014 | PANSS-T |  | NSA-16 |  |
| Jarskog 2015 | BPRS-T |  | SANS |  |
| Othman 2014 |  |  |  |  |
| Poyurovsky 2013 |  | SAPS | SANS | HDRS |
| Smith 2018a | BPRS-T |  |  |  |
| Smith 2018b |  |  |  |  |
| Wang 2021 | PANSS-T | PANSS-P | PANSS-N |  |
| NCT00690274 2019 |  |  |  | MADRS |

† For Egan 2013 study, the working memory was assessed using BACS (Digit Sequencing tasks) and CNB (N-back)..

BACS: Brief Assessment of Cognition in Schizophrenia, BPRS-T: Brief Psychiatric Rating Scale-total score, BVMT-R: Brief Visuospatial Memory Test-Revised CNB: University of Pennsylvania Computerized Neuropsychological battery, HDRS: Hamilton Depression Rating Scale, MADRS: Montgomery Åsberg Depression Rating Scale, MCCB: The Measurement and Treatment Research to Improve Cognition in Schizophrenia Consensus Cognitive Battery, NSA-16: 16-item Negative Symptom Assessment Scale, PANSS (-T, -P, -N): Positive and Negative Syndrome Scale (-total score, -positive subscale’s score, -negative subscale’s score), SANS: Scale for the Assessment of Negative Symptoms, SAPS: Scale for the Assessment of Positive Symptoms,

**Table S3. The results of subgroup analysis including studies on betahistine**

**S3.1. Continuous variable**

|  | **Subgroup including BET studies and BET + REB study** | | | | | | **Subgroup including only BET studies** | | | | | |
| --- | --- | --- | --- | --- | --- | --- | --- | --- | --- | --- | --- | --- |
| **Outcome** | **K** | **n** | **SMD** | **95% CI** | ***p*** | **Heterogeneity** | **K** | **n** | **SMD** | **95% CI** | ***p*** | **Heterogeneity** |
| Composite scores of the cognitive tests | 1 | 89 | −0.61 | −1.03, −0.18 | **0.01** | na | 1 | 89 | −0.61 | −1.03, −0.18 | **0.01** | na |
| MCCB speed of the processing scores | 1 | 89 | −0.44 | −0.87, −0.02 | **0.04** | na | 1 | 89 | −0.44 | −0.87, −0.02 | **0.04** | na |
| MCCB attention/vigilance scores | 1 | 89 | −0.43 | −0.85, −0.01 | **0.04** | na | 1 | 89 | −0.43 | −0.85, −0.01 | **0.04** | na |
| Working memory scores | 1 | 89 | −0.48 | −0.90, −0.06 | **0.02** | na | 1 | 89 | −0.48 | −0.90, −0.06 | **0.02** | na |
| MCCB verbal learning scores | 1 | 89 | −0.62 | −1.04, −0.19 | **0.00*** | na | 1 | 89 | −0.62 | −1.04, −0.19 | **0.00*** | na |
| Visual learning scores | 1 | 89 | −0.57 | −1.00, −0.15 | **0.01** | na | 1 | 89 | −0.57 | −1.00, −0.15 | **0.01** | na |
| MCCB reasoning/problem solving scores | 1 | 89 | −0.41 | −0.83, 0.01 | 0.06 | na | 1 | 89 | −0.41 | −0.83, 0.01 | 0.06 | na |
| MCCB social cognition scores | 1 | 89 | −0.28 | −0.69, 0.14 | 0.20 | na | 1 | 89 | −0.28 | −0.69, 0.14 | 0.20 | na |
| Total symptom scores | 4 | 249 | −0.11 | −0.36, 0.14 | 0.38 | *I^2^* = 0.00%, | 4 | 249 | −0.11 | −0.36, 0.14 | 0.38 | *I^2^* = 0.00%, |
| Positive symptom scores | 3 | 226 | −0.22 | −0.48, 0.05 | 0.11 | *I^2^* = 0.00% | 2 | 183 | −0.25 | −0.54, 0.04 | 0.09 | *I^2^* = 0.00% |
| Negative symptom scores | 3 | 226 | −0.11 | −0.38, 0.15 | 0.39 | *I^2^* = 0.00% | 2 | 183 | −0.16 | −0.45, 0.13 | 0.28 | *I^2^* = 0.00% |
| PANSS general subscale scores | 2 | 183 | 0.12 | −0.17, 0.41 | 0.42 | *I^2^* = 0.00% | 2 | 183 | 0.12 | −0.17, 0.41 | 0.42 | *I^2^* = 0.00% |
| Depressive symptom scores | 1 | 43 | −4.04 | −5.10, −2.97 | **0.00**** | na |  |  |  |  |  |  |
| CGI-S scores | 1 | 43 | −0.20 | −0.84, 0.44 | 0.55 | na |  |  |  |  |  |  |
| Body mass index | 3 | 176 | −0.33 | −1.00, 0.34 | 0.34 | *I^2^* = 76.27% | 2 | 133 | 0.03 | −0.31, 0.37 | 0.85 | *I^2^* = 0.00% |
| Body weight | 3 | 176 | −0.28 | −0.96, 0.39 | 0.41 | *I^2^* = 76.66% | 2 | 133 | 0.07 | −0.27, 0.41 | 0.67 | *I^2^* = 0.00% |
| Waist circumference | 2 | 133 | 0.13 | −0.21, 0.47 | 0.45 | *I*^2^ = 0.00% | 2 | 133 | 0.13 | −0.21, 0.47 | 0.45 | *I*^2^ = 0.00% |
| Hip circumference | 2 | 133 | 0.18 | −0.16, 0.52 | 0.31 | *I*^2^ = 0.00% | 2 | 133 | 0.18 | −0.16, 0.52 | 0.31 | *I*^2^ = 0.00% |
| Serum triglyceride levels | 2 | 122 | −0.15 | −0.51, 0.20 | 0.41 | *I*^2^ = 0.00% | 2 | 122 | −0.15 | −0.51, 0.20 | 0.41 | *I*^2^ = 0.00% |
| Serum LDL cholesterol levels | 2 | 122 | −0.09 | −0.45, 0.26 | 0.60 | *I*^2^ = 0.00% | 2 | 122 | −0.09 | −0.45, 0.26 | 0.60 | *I*^2^ = 0.00% |
| Serum total cholesterol levels | 2 | 122 | 0.01 | −0.34, 0.37 | 0.95 | *I*^2^ = 0.00% | 2 | 122 | 0.01 | −0.34, 0.37 | 0.95 | *I*^2^ = 0.00% |

**S3.2. Dichotomous variable**

|  | **Subgroup including BET studies and BET + REB study** | | | | | | **Subgroup including only BET studies with reboxetine** | | | | | |
| --- | --- | --- | --- | --- | --- | --- | --- | --- | --- | --- | --- | --- |
| **Outcome** | **K** | **n** | **RR** | **95% CI** | ***p*** | **Heterogeneity** | **K** | **n** | **RR** | **95% CI** | ***p*** | **Heterogeneity** |
| All-cause discontinuation | 5 | 280 | 0.84 | 0.45, 1.58 | 0.59 | *I^2^* = 21.25% | 4 | 237 | 0.76 | 0.31, 1.88 | 0.56 | *I^2^* = 41.61% |
| Anorexia/decreased　appetite/loss of appetite | 2 | 128 | 1.23 | 0.48, 3.15 | 0.67 | *I^2^* = 0.00% | 2 | 128 | 1.23 | 0.48, 3.15 | 0.67 | *I^2^* = 0.00% |
| Tremor | 3 | 164 | 0.94 | 0.36, 2.46 | 0.89 | *I^2^* = 11.31% | 3 | 164 | 0.94 | 0.36, 2.46 | 0.89 | *I^2^* = 11.31% |
| Nausea | 2 | 128 | 1.01 | 0.31, 3.31 | 0.99 | *I^2^* = 0.00% | 2 | 128 | 1.01 | 0.31, 3.31 | 0.99 | *I^2^* = 0.00% |
| Constipation | 2 | 128 | 0.45 | 0.13, 1.57 | 0.21 | *I^2^* = 58.13% | 2 | 128 | 0.45 | 0.13, 1.57 | 0.21 | *I^2^* = 58.13% |
| Increased blood levels of antipsychotics | 2 | 183 | 2.97 | 0.31, 28.01 | 0.34 | *I*^2^ = 0.00% | 2 | 183 | 2.97 | 0.31, 28.01 | 0.34 | *I*^2^ = 0.00% |

* 0.004

** < 0.000000001

95% CI: 95% confidence interval, CGI-S: Clinical Global Impressions-Severity, K: number of studies, LDL: low-density lipoprotein, MCCB: The Measurement and Treatment Research to Improve Cognition in Schizophrenia Consensus Cognitive Battery, n: number of individuals, na: not applicable, PANSS: Positive and Negative Syndrome Scale, RR: risk ratio, SMD: standardized mean difference
